# Non‐Invasive Imaging for the Evaluation of a New Oral Supplement in Skin Aging: A Case‐Controlled Study

**DOI:** 10.1111/srt.70171

**Published:** 2025-05-24

**Authors:** Simone Michelini, Maria Elisabetta Greco, Giordano Vespasiani, Federica Trovato, Camilla Chello, Noah Musolff, Carmen Cantisani, Giovanni Pellacani

**Affiliations:** ^1^ Dermatology Clinic Department of Clinical Internal Anesthesiological and Cardiovascular Sciences University of Rome, “Sapienza” Rome Italy; ^2^ Dermatology Department Istituto Dermopatico dell'Immacolata, IDI‐IRCCS Rome Italy

**Keywords:** antioxidant, collagen, food supplement, inflammatory aging, skin aging, wrinkle

## Abstract

**Background:**

Skin aging represents a para‐physiological process related to metabolic imbalances, inflammation, proliferative responses, and oxidative stress. Ending the cycle of inflammation, and oxidative stress represents a way to slow the effects of aging.

**Study Objective:**

The aim of our study was to evaluate the benefits of supplements with an herbal mixture based on Venerinase and B‐group vitamins, Zinc, and Magnesium in preventing/reducing photoaging by means of objective quantitative analysis of visual skin features obtained by VISIA 2D and stroma and vascular characteristics measured by Optical Coherence Tomography (OCT).

**Methods:**

A prospective clinical study was conducted in 40 participants. They were randomized 1:1 to receive the supplement (Giovina) or placebo. Assessments were conducted at Baseline (T0), after 2 months (T1), and after 4 months (T2). The primary endpoint was to obtain a significant improvement in facial photo/chrono‐aging in the treatment arm, as assessed through the evaluation of the Griffith scale. Secondary endpoints were to assess significant changes in skin aging objective features (VISIA) and Optical Coherence Tomography (OCT) parameters.

**Results:**

The treated group showed a significant reduction in the mean Griffith scale score from 4.5 at baseline to 3.76 at T2 (4 months) (*p* = 0.001). A significant decrease in red areas and a borderline significant reduction in the number of wrinkles have been shown by skin aging objective feature calculation on VISIA 2D photographs. OCT analysis showed a borderline significant reduction in collagen density.

**Conclusions:**

The decrease in vascular pattern and collagen density suggests an anti‐inflammatory effect with a potential stromal remodeling in the treated group. This seems to be correlated to the reduction in wrinkles and facial redness.

## 1 Introduction

Skin aging is a multi‐factorial, para‐physiological process, resulting from endogenous and exogenous factors that lead to changes in the cellular and stromal organization of the skin. Typically, we distinguish between extrinsic and chronological (or intrinsic) aging [1]. Extrinsic aging is caused by external environmental agents, with Ultra‐Violet Rays (UVR) being the most relevant. The changes linked to extrinsic aging include elastosis, dyspigmentation, and keratinocyte dyskeratosis [2, 3, 4]. Among the factors that induce aging alterations, chronic inflammation should be considered an important contributor, generating epidermal and stromal damage defined in the so‐called “inflamm‐aging” [5, 6, 7]. The aging process, which is characterized by a gradual deterioration of interconnected physiological systems and metabolic pathways, can exacerbate immune system impairment and increase susceptibility to inflammatory diseases [8]. Due to its association with numerous degenerative illnesses, inflamm‐aging is a relatively recent notion that emphasizes an age‐related low level of uncontrolled chronic inflammatory condition has emerged as a promising area of study [5, 9–11]. Sun‐exposure, pollution, topical application of irritants and chemicals, and other environmental factors represent important stressors for the skin [2, 5, 12]. UVR exposure is thought to be responsible for about 80% of skin aging [13]. Furthermore, epidemiological research has demonstrated a strong correlation between photoaging and a number of skin conditions, such as melanoma, basal cell carcinoma, actinic keratosis, and elastosis [14]. The primary cause of intrinsic aging is the loss of metabolic efficiency and the tissue's capacity to regenerate its stromal and cellular components, which leads to gradual thinning and a reduction in firmness [1, 15]. Moreover, during menopause, a significant part of the visible skin changes depends on the progressive reduction in sex hormone production [16, 17, 18, 19, 20]. The epidermis becomes thinner and dehydrated, while the dermis loses collagen. The most evident consequence is thinned, xerotic skin that loses tone and elasticity [21, 22]. In the last decades there has been an increase of patients requiring cosmetic procedures to improve wrinkles, laxity, and changes in pigmentation [23, 24] and more and more attention has been focused on oral supplements that can counteract and improve the metabolic and structural alteration of skin aging [13, 25, 26]. This growing interest is driven by the easier handling of a supplement in comparison to the more invasive surgical and injectable procedures [26, 27]. Numerous oral supplements to prevent skin aging have been proposed, such as niacinamide, melatonin, vitamins, glucosamine, glutathione, collagen, hyaluronic acid, zinc, and so forth, in the treatment of skin aging [27, 28, 29, 30, 31]. The scarcity of systematic clinical studies, except for collagen and hyaluronic acid oral supplementation [28, 32], and the insufficient evidence do not support the use of food supplements to reduce the effects of aging [28]. A new product has been recently developed (Giovina), based on the combination of ingredients active on skin aging‐related mechanisms, namely Venerinase (an extract of rhodiola rosea, tribulus terrestris, moringa oleifera, undaria pinnatifida), folic acid, and vitamins B1, B2, B6, B12, and magnesium [21, 33, 34]. Native to central Europe and Asia, Rhodiola rosea Lam. is a medicinal plant with adaptogenic qualities, known in the literatures and demonstrated by clinical research [33, 35, 36]. The phenolic chemicals, terpenes and rosavins that belong to the class of phenylpropanoids, specific metabolites of the root and rhizome, have been partially linked to the pharmacological characteristics of R. rosea extracts [37, 38]. Tribulus terrestris L. is a tropical plant in the Zygophyllaceae family, native to Asia and Africa [21, 39]. The presence of a high concentration of saponins is believed to be partly responsible for its various properties, including the ability to effectively combat oxidative stress and inflammation, so much so that it is proposed in the diets of runners and athletes [37]. The medicinal plant Moringa oleifera Lam., is a perennial plant belonging to the Moringaceae family, has long been used to treat various inflammatory and metabolic conditions [37, 40, 41]. Its leaves appear to have neuroprotective effects, due to a high concentration of phenolic chemicals, which could be the key to the results obtained from M. oleifera extracts in preclinical models of neurotoxicity. This could occur through restoration of the mitochondrial respiratory chain [40, 41]. In addition, a recent preclinical investigation in mice showed that ethanol leaf extract has anti‐fatigue qualities [37]. Presumably due to the presence of fucoxanthin, the primary bioactive component, Undaria pinnatifida, also known as wakame, a seaweed in the Alariaceae family, has been shown to have excellent neuroprotective properties [42, 43]. Wakame also contains phenolic compounds and flavonoids, implying that the neuroprotective effects of the seaweed may be the result of modulating different antioxidant and anti‐inflammatory pathways [37, 44].

Despite the pre‐clinical results may lead to the claim that the product could be an effective supplement to counteract skin aging, there is no clinical evidence that its use is able to lead to visible improvement after a short time of supplementation.

Our study aims to evaluate whether the systematic use of the dietary supplement Giovina can improve the appearance of aged skin, giving a more youthful look. We conducted a randomized trial, using non‐invasive technologies to objectively measure the benefits of Venerinase implementation on skin aging, compared to a control group. The primary endpoint of our study is to demonstrate a significant reduction in skin aging score in the treated group after 4 months of supplementation. Secondary endpoints are the reduction of skin aging objective parameters obtained by VISIA standardized photography and D‐OCT in order to understand the compartments of action and to objectively prove its effects.

## 2 Materials and Methods

This study is a prospective, controlled, randomized, single‐blind, two‐arm (treatment vs. control) clinical trial. Forty healthy and immunocompetent females aged between 40 and 60 years were enrolled at the dermatology clinic of Policlinico Umberto I of Rome, from May to December 2022. Inclusion criteria were the presence of mild/moderate aging (3–5 in the Griffith aging scale). Exclusion criteria included very mild or severe skin aging (Griffith aging scale <3; >6, respectively), previous or concomitant diagnosis of skin cancer on the face or scalp, tumors of any nature that may have systemic involvement, previous diagnosis of chronic or active dermatological conditions that could affect the skin aspect or structure, pregnant or breastfeeding women, subjects currently undergoing menopause treatment (Hormone Replacement Therapy—HRT or supplements) or receiving any other systemic or topical therapies for chronic inflammatory diseases, individuals who had topical or subcutaneous anti‐aging therapies both during and in the month before the study, subjects with any contraindications or allergies to elements/excipients present in the study products and subjects unable to understand the consent or deemed unfit to follow the study instructions.

Enrolled subjects were randomized 1:1, and those in the treatment arm received the supplement (Giovina) with specific instructions to take throughout the study duration. The indication was to assume two sachets (4,5 g × 2) every day for the entire study duration. The control arm followed the regular visits and assessments schedule without any food supplement supply.

At the baseline visit (T0) all subjects signed the informed consent, and subject characteristics were recorded as per the inclusion/exclusion criteria checklist. Furthermore, Fitzpatrick skin phototype, occupational and sun exposure history, smoking and alcohol use, and BMI were recorded as possible confounding factors.

The Griffith's scale for clinically rating the subject's level of aging from 0 to 9 (15) was evaluated by a blind investigator, unaware of randomization assignation, at baseline (T0), after 60 ± 7 days (T1), and after 120 ± 7 days (T2) visualizing the standardized clinical pictures carried out by means of Canfield VISIA system model Generation 7 (Canfield Scientific, Parsippany, NJ, USA) as per the study protocol.

Instrumental assessments were conducted at baseline (T0), after 60 ± 7 days (T1), and after 120 ± 7 days (T2) consisting of standardized imaging acquisition of the face in three projections (frontal, left, and right 45° projections) by means of Canfield VISIA system model Generation 7 and by in vivo microscopic assessment of the epidermis and stroma by means of high‐definition Dynamic‐Optical Coherence Tomography (D‐OCT) (Vivosight, Michelson Diagnostics).

From VISIA images, objective measures were automatically obtained by means of a dedicated software for the evaluation of wrinkle extent (WRINKLE), pigmentation (BROWNSPOT), erythema (REDSPOT), and the presence of porphyrins (PORPHIRIN), calculated on a standard mask and reported as absolute values (ABS) and object count (COUNT).

Concerning D‐OCT imaging, a target area of 1.6 × 1.6 mm^2^ on the right cheek, zygomatic area, was selected at baseline and marked on transparent film in order to repeat the measure approximately in the same area at follow‐up visits. Objective parameters were extracted from the images using the dedicated VivoSight software, including epidermal thickness, collagen density, collagen intensity attenuation, and vascular intensity at 150‐, 300‐, and 500‐microns depth, as described in the literature.

During each follow‐up visit, any adverse event was reported. It has been ensured that the study was conducted in accordance with Good Clinical Practice (GCP) guidelines, ethical principles stated in the Declaration of Helsinki, and current regulations for observational studies. The study was approved by the local Ethical Committee (prot. N. 1034/2021, V1, June 29, 2021)

Before participating in the study, subjects were provided with detailed information about the study objectives and the methods used to achieve them through a specific and detailed Informed Consent Form. The Informed Consent Form was given to eligible subjects, and only after receiving all necessary information, the subjects provided their informed consent in a completely free and voluntary manner to be included in the study. All paper documents, including informed consents, were kept in a secure location, ensuring the privacy and confidentiality of the participants' data.

### 2.1 Data Storage and Privacy

The data were collected anonymously, with no possibility for researchers to trace the identities of participating subjects, and were adequately recorded in a computerized database, which also does not contain any personal identifiers.

### 2.2 Statistical Analysis

The collected data were described in terms of percentages for categorical variables and standard deviations for continuous variables. The results were tested regarding their association with outcome measures as follows: for non‐parametric measures, the comparisons between baseline (T0), intermediate (T1), and final (T2) measures were performed through the Kruskal–Wallis test and the Mann–Whitney U test. Continuous variables were evaluated using a paired *t*‐test and ANOVA. Comparisons between treated subjects and controls at different time points were analyzed using *t*‐tests for parametric data and chi‐square tests for non‐parametric data. A *p* < 0.05 was considered significant.

### 2.3 Sample Estimation

The research hypothesis aimed to improve the treatment of skin aging through the use of supplements, based on preliminary results indicating an in vitro action capable of inhibiting inflammatory processes associated with chronic damage to collagen and epidermis, responsible for typical manifestations of aging, such as wrinkles, sagging, thinning of the epidermis, and so forth. Due to limited knowledge about the specific supplement's action, the sample size calculation was challenging, and therefore, the study has been considered a “pilot study” to provide more accurate information on the supplement's action in the future.

## 3 Results

At baseline, there were no statistically significant differences between the control group and treatment group in terms of age (mean age: 52 years old vs. 54 years old), Griffith scale aging score, or other factors.

In relation to the primary endpoint, statistical analysis revealed a substantial improvement in the treated group's Griffith scale aging score, which decreased from an average of 4.5 at baseline to 3.8 at T2 (*p* = 0.001). In the control group, no significant difference was seen, as we can see in Table 1.

**TABLE 1:** Statistical analysis revealed a substantial improvement in the treated group's Griffith scale aging score. In the control group, no significant difference was seen.

	CTRL			Treatment		
Parameters	Mean	SD	*p*	Mean	SD	*p*
AGING SCORE_T0	4.7	0.865		4.5	1.15	
AGING SCORE_T1	4.9	0.968	0.104	4.39	1.092	0.163
AGING SCORE_T2	4.9	0.968	0.104	3.76	0.903	0.001**

**FIGURE 1:**
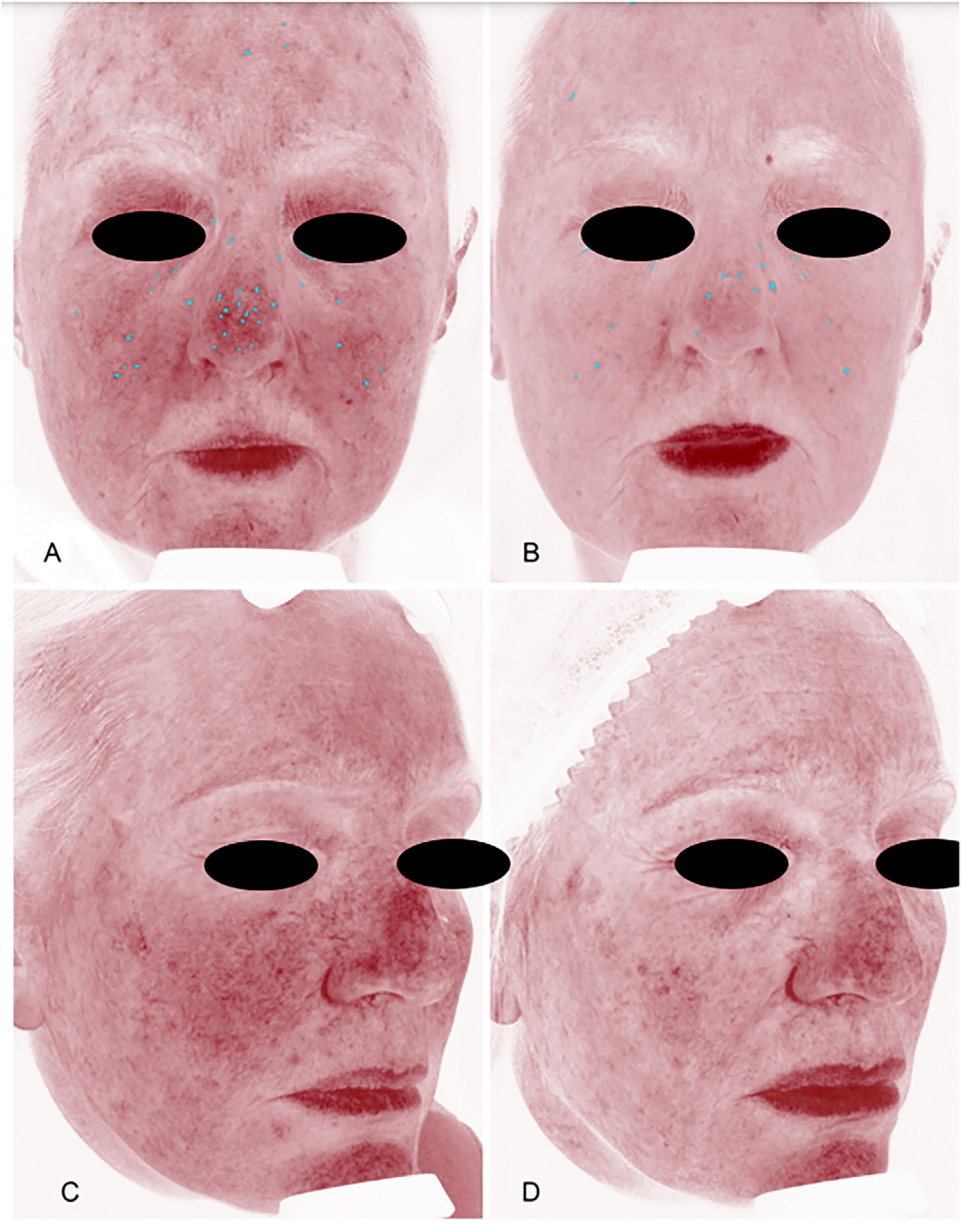
VISIA *system 2D photographs* show a significant reduction in the treatment group for the extent of erythema at T2 (B–D) compared with T0 (A–C) in two different patients. The significant reduction in parameters related to erythema may be attributed to the anti‐inflammatory action of the active ingredients in the treatment group.

VISIA system parameters also showed a significant reduction in the treatment group for the extent of erythema (Redspot_Abs) (*p* = 0.004), and number of red areas (Redspot_Count) (*p* = 0.021) (Figure 1).

**FIGURE 2:**
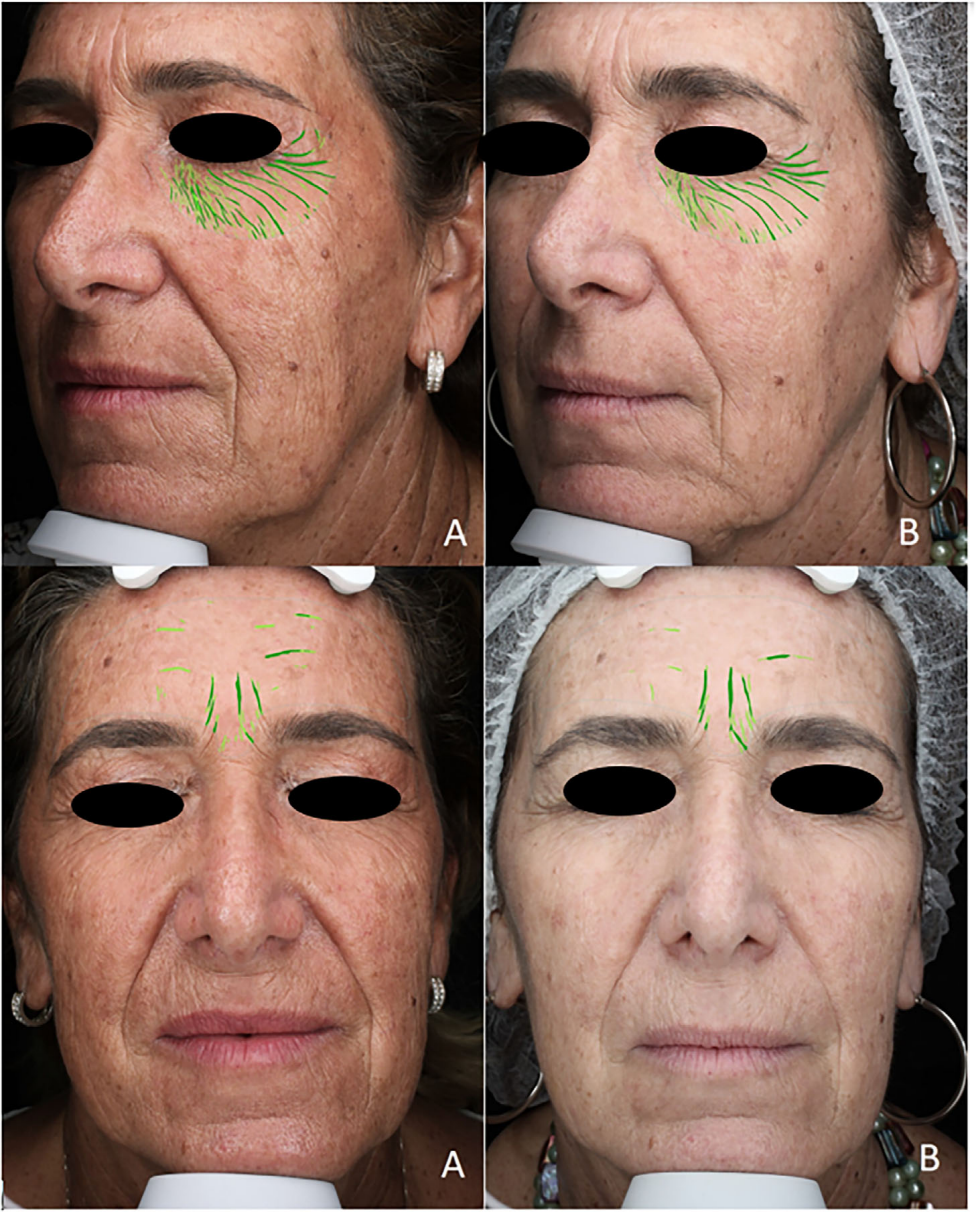
Visible significant improvement in Visia 2D images on aging score according to the Griffith scale at T0 (A) and T2 (B). Visia software shows objective reduction of visible wrinkles (green lines).

Additionally, there was a 23% decrease in the number of visible wrinkles (Wrinkle_count) in the treated patients (*p* = 0.059) at T2, compared to a 10% decrease in the control group (Figure 2).

Untreated subjects showed an increase in the value of porphyrins compared to baseline, both in terms of extent (Porph_Abs) (*p* = 0.042) and the number of spots (Porph_Count) (*p* = 0.052) at T2, as we can see in Table 2.

**TABLE 2:** Clinical parameters observed in Visia 2D images of the control group and treated subjects.

	CTRL			Treatment		
Parameters	Mean	SD	*p*	Mean	SD	*p*
WRINKLE_ABS_T0	16 817	10 493.559		23 110.53	14 636.518	
WRINKLE_ABS_T1	17 404.27	7594.11	0.843	21 940.47	15 299.526	0.714
WRINKLE_ABS_T2	17 123.55	11 176.398	0.700	18 538.53	13 406.084	0.174
WRINKLE_COUNT_T0	44.6	20.357		50.33	27.515	
WRINKLE_COUNT_T1	41.27	20.19	0.481	46.6	23.925	0.522
WRINKLE_COUNT_T2	40.36	35.138	0.881	38.53	20.743	**0.059**
BROWNSPOT_ABS_T0	26 147.93	8340.813		23 743.4	6964.362	
BROWNSPOT_ABS_T1	27 720.13	7576.875	0.218	24 802.87	5579.333	0.407
BROWNSPOT_ABS_T2	25 552.36	6047.202	0.803	23 339.36	6052.047	0.902
BROWNSPOT_COUNT_T0	329.53	124.889		290.6	92.999	
BROWNSPOT_COUNT_T1	341	100.766	0.466	318.87	86.752	0.178
BROWNSPOT_COUNT_T2	321.09	102.558	0.976	296.87	95.21	0.775
REDSPOT_ABS_T0	17 540.4	5623.539		15 500.07	5480.572	
REDSPOT_ABS_T1	17 531.73	6095.37	0.992	15 868.07	5745.805	0.804
REDSPOT_ABS_T2	16 541.27	4396.612	0.391	12 848.79	4247.78	**0.004** **
REDSPOT_COUNT_T0	77.33	39.55		57.67	35.862	
REDSPOT_COUNT_T1	79.73	40.086	0688	62	30.759	0.551
REDSPOT_COUNT_T2	71.18	37.823	0.445	45.67	29.374	**0.021** *
PORPH_ABS_T0	14 092.93	9086.587		15 549.33	9849.516	
PORPH_ABS_T1	16 167.86667	12 523.02025	0.471	15 180.36573	10 031.05811	0.884
PORPH_ABS_T2	22 819.18	14 561.964	**0.042** *	13 923.87	9433.962	0.290
PORPH_COUNT_T0	2249	1495.785		2527.47	1546.803	
PORPH_COUNT_T1	2533.87	1923.297	0.544	2480.4	1593.919	0.912
PORPH_COUNT_T2	3615.73	2305.811	**0.052**	2214.53	1444.991	0.221

**p < 0.001

*p < 0.01

**FIGURE 3:**
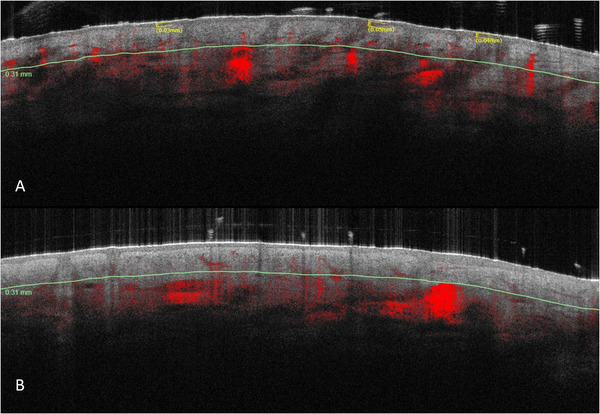
OCT image shows a mild reduction in collagen density in the treated subjects (A) compared to controls (B). Vascular parameters show no significant variation.

Changes in D‐OCT parameters related to skin aging showed a reduction in collagen density in the treated subjects compared to controls with borderline significance (*p* = 0.059), but no statistical differences in vascular extent and epidermal thickness, as we can see in Table 3 and Figure 3.

**TABLE 3:** Changes in D‐OCT parameters related to skin aging. It shows a reduction in collagen density in the treated subjects compared to controls with borderline significance (*p* = 0.059), but no statistical differences in vascular extent and epidermal thickness.

	CTRL			Treatment		
Parameters	Mean	SD	*p*	Mean	SD	*p*
EPIDTHIC_T0	0.0467	0.00866		0.0583	0.02137	
EPIDTHIC_T1	0.0411	0.00782	0.179	0.045	0.00548	0.221
EPIDTHIC_T2	0.0429	0.01254	0.356	0.0467	0.00888	0.127
COLLDENS_T0	60.11	7.288		66.43	5.503	
COLLDENS_T1	54.78	15.522	0.224	56.29	11.191	0.089
COLLDENS_T2	57.57	14.105	0.839	47.67	11.428	0.059
COLLATT_T0	0.0256	0.00527		0.0214	0.0069	
COLLATT_T1	0.0222	0.00833	0.195	0.0243	0.00535	0.457
COLLATT_T2	0.0243	0.00535	0.172	0.0225	0.00622	0.754
VASC150_T0	1316.83	479.608		1670.75	536.255	
VASC150_T1	1131.33	291.228	0.179	1298.25	650.871	0.326
VASC150_T2	2045.5	1931.783	0.498	1325.71	522.167	0.332
VASC300_T0	18 249.57	10 673.586		14 03875	8007.041	
VASC300_T1	13 652.71	7036.646	0.173	9240.25	4053.793	0.401
VASC300_T2	18 716.75	4671.118	0.839	12 309	5458.413	0.403
VASC500_T0	25 607.67	19 937.05		25 857	11 711.902	
VASC500_T1	19 203.33	14 431.121	0.339	12 201.75	13 797.843	0.334
VASC500_T2	18 877.67	13 683.843	0.163	21 359	9732.87	0.498

The primary objective has been achieved, demonstrating that the use of the product leads to a reduction in the median aging score in the treated group, while no significant variation is observed in the control group compared to baseline.

The secondary objectives show that the use of the product results in a reduction in the number of WRINKLES (a factor strongly correlated with the perception of age/aging) by approximately 12 points, compared to 7 points in the control group. Additionally, the significant reduction in parameters related to ERYTHEMA can be attributed to the anti‐inflammatory action of the active ingredients in the treatment.

The increase in porphyrins observed in the control group can be explained by seasonal factors, with a higher prevalence of subjects recruited during the summer and evaluated in the autumn when porphyrins (associated with C. Acne activity) are typically inhibited due to sun exposure, while being more expressed during seasonal changes and in autumn. The maintenance of porphyrin signal intensity in the treated group can, therefore, be interpreted as the product's ability to maintain a state of cutaneous eubiosis, either through direct action on C. Acne or indirectly by limiting local inflammatory processes and modifying cutaneous homeostasis and barrier conditions, leading to a healthy skin balance.

The decrease in collagen density can be justified by increased hydration of the stroma, which is compatible with a reduction in wrinkles and perceived aging. This can be attributed to a higher production of hyaluronic acid and other glycosaminoglycans, neo‐collagen, and/or a reduction in elastosis. These factors should be further investigated in the future through specific clinical laboratory studies.

## 4 Discussion

This study delves into the objective evaluation of clinical and anatomical modification induced by a specific food supplement prepared for counteracting skin aging. The product is based on a botanical active mix based on Venerinase (rhodiola rosea, tribulus terrestris, moringa oleifera, undaria pinnatifida), folic acid, vitamin B1, B2, B6, B12, zinc, and magnesium. The systematic use of supplements containing ingredients with anti‐inflammatory and antioxidant mechanisms in chronic low‐grade inflammation is supported by studies in the literature [33, 34, 45].

The skin, which is continually subjected to numerous irritants, serves as the host's contact with the environment, together with other tissues [46]. UV radiation exposure plays a primary role in skin aging. UV (primarily UV‐B radiation) affects the skin through the creation of pyrimidine‐pyrimidine dimers, causing DNA damage, harmful effects on the extracellular matrix, inflammation, and immunosuppression, and subsequent oxidative stress [8, 47, 48]. The generation of arachidonic acid and the oxidation of membrane lipids are the causes of UV's inflammatory action. Arachidonic acid is converted into prostaglandins by cyclooxygenase enzymes (COX), which increases the recruitment of inflammatory cells and the production of reactive oxygen species (ROS), interleukin‐6 (IL‐6), and other pro‐inflammatory cytokines [48, 49, 50]. Additionally, ROS also induces the activation of tumor necrosis factor‐α (TNF‐α) and NF‐κB expression. Furthermore, UV increases ROS‐induced peroxidation, which triggers the development of inducible nitric oxide synthase, an enzyme that regulates angiogenesis and hyperpermeability through the overexpression of VEGF (vascular endothelial growth factor) [51]. Both intrinsic factors, such as hormonal changes during menopause, and extrinsic factors like chronic inflammation induced by environmental stressors play a major role in this process [52]. In particular, the increase in cortisol can lead to skin imperfections due to metabolic and oxidative distress caused by highly reactive oxygen species known as free radicals or ROS (Reactive Oxygen Species), which attack cellular constituents such as membranes, enzymes, and DNA, causing damage [53].

To preserve homeostasis under inflammatory stresses, many immune cell types are either resident in or recruited into the skin. Notably, senescent cells accumulating in the skin during aging have a primary role in driving skin inflamm‐aging, exhibiting the specific downregulation of histone deacetylase (HDAC), inducing the appearance of senescence biomarkers in dermal fibroblasts and consequent dermal fibrosis [54, 55]. Therefore, it is believed that one of the most effective actions that can be taken to slow down the effects of aging is to find ways to break free from the cycle of inflammation and oxidative stress. Numerous substances, such as antioxidants, vitamins, and so forth, are used and recommended for skin wellbeing and to counteract skin aging, both when applied topically and as dietary supplements [56, 57].

From the results obtained in preclinical analysis, Venerinase showed a valid anti‐inflammatory action by the modulation of the two early fibrosis factors, HDAC4 and SPARC, and reducing the expression of genes associated with inflamm‐aging, specifically TNFα and IL‐6 [58].

The study's methodology aimed to objectively demonstrate that the in vitro findings match with clinically visible results.

The clinical assessment was based on the Griffith score evaluated in a blind from collected standardized images in order to avoid observer selection bias. The Griffith score showed a significant improvement after 4 months in the group receiving food supplementation, whereas it remained stable in the control group. This clinical finding found objective demonstration in operator‐independent standardized clinical image analysis obtained with the Canfield VISIA system. A significant reduction was observed in the treatment group for skin erythema parameters, suggesting an evident anti‐inflammatory action (Figure 1).

Although borderline, a decrease in the number of visible wrinkles was measured in the treatment group. Concerning the underlying anatomy of the skin, as evaluated by D‐OCT, a moderate effect on the stromal structure was observed, consisting of a borderline significant reduction of collagen density. This finding, correlated with wrinkle count reduction and skin quality improvement, may be explained by the enrichment of the matrix components, leading to greater hydration of the stroma and thus an increased homogeneity of the D‐OCT signal.

The increase in porphyrin levels among untreated subjects presents an interesting dilemma. It is known that porphyrin expression is related to skin microbiome alterations, especially linked to C. acne [59]. This outcome prompts crucial questions about the supplement's interaction with specific skin components and microenvironment, and we may hypothesize that the trans‐seasonal duration of the study (starting in spring and ending in late autumn and wintertime) probably interfered in the porphyrin presence in the untreated group. However, if this is demonstrated, we can suppose that the Giovina food supplement may also act as a microenvironment modulator, which can be beneficial for sebum production and microbic balance.

Study limitations are represented by a small sample, trans‐seasonal conduction, limited age ranges, and only female sex. Also, the study could benefit from a more detailed exploration of the supplement's underlying mechanisms in order to provide valuable insights into its mode of action at the cellular level. However, this study represents a unique objective evaluation of the effect of a food supplement on skin aging parameters.

## 5 Conclusions

This study combines clinical blind evaluations with objective instrumental and in vivo microscopic analyses to assess clinical effects. The documented anti‐inflammatory action is highlighted by the objective reduction in erythema, the clinical parameter mostly correlated with inflammatory aging. Moreover, in D‐OCT analysis, the treated group's decreased collagen density suggests possible stromal remodeling, the consequence of a reduction in visible and measurable wrinkles. Altogether, this results in a decreased perception of the subject's aging, as measured by clinical score. These findings are in line with previous in vitro experiments that showed the antioxidant and metabolic capabilities of the product.

This study measured evident clinical effects in the relatively short term, demonstrating how the use of a supplement could lead to actual visible clinical benefits based on its biological effects on skin anatomy. Further studies are required to consolidate these results. However, the use of objective measures from standardized technologies seems to represent an innovative methodological approach to better evaluate product effects on skin aging.

## Conflicts of Interest

The authors certify that there is no conflict of interest with any financial organization regarding the material discussed in the manuscript.

## Data Availability

The data that support the findings of this study are available from the corresponding author upon reasonable request.
